# Supplementary material for: Microencapsulation of Flaxseed Oil by Lentil Protein Isolate-κ-Carrageenan and -ι-Carrageenan Based Wall Materials through Spray and Freeze Drying
Source: Molecules. 2022 May 17;27(10):3195. doi: 10.3390/molecules27103195 (PMC9145131; doi:10.3390/molecules27103195)
Supplement: Supplementary file 1 [file molecules-27-03195-s001.zip › molecules-1469919-supplementary.pdf]

Table S1. Proximate composition of all biopolymers used in this research. Data represents the mean ± one standard deviation (n = 3). All data is reported as the percentage on a dry weight basis.

Materials	Protein[1]	Lipid	CHO[2]	Ash
Lentil protein isolate	80.6 ± 0.3	0.8 ± 0.1	13.0	5.6 ± 0.4
κ-Carrageenan	Nil	Nil	77.6	22.4 ± 0.2
ι-Carrageenan	Nil	Nil	71.2	28.8 ± 0.2
Maltodextrin	Nil	Nil	99.3	0.7 ± 0.0

Notes:

[1]Nitrogen conversion factor (6.25)

[2]Carbohydrate (CHO) was determined based on the dry weight difference 100% and (protein + ash + lipid) levels.

Table S2. Formulations used for the initial emulsions to deliver 20% and 30% oil within microcapsules with both types of lentil protein isolate-carrageenan (LPI-CAR) formulations.

Sample	%final oil	Initial emulsions (%, w/w)				Spray dried microcapsules (%, w/w)				
		% oil	LPI/ LPI-ι-C/ LPI-κ-C	MD	total solid	core: wall	oil	LPI/ LPI-ι-C/ LPI-κ-C	MD	total solid
LPI 20%	20	5	1	19	25	1:4	20	4	76	100
LPI 30%	30	7.5	1	16.5	25	1:2.3	30	4	66	100
LPI-ι-C 20%	20	5	1	19	25	1:4	20	4	76	100
LPI-ι-C 30%	30	7.5	1	16.5	25	1:2.3	30	4	66	100
LPI-κ-C 20%	20	5	1	19	25	1:4	20	4	76	100
LPI-κ-C 30%	30	7.5	1	16.5	25	1:2.3	30	4	66	100

- The percentage on the name of the samples represents the final oil content in dried powders.

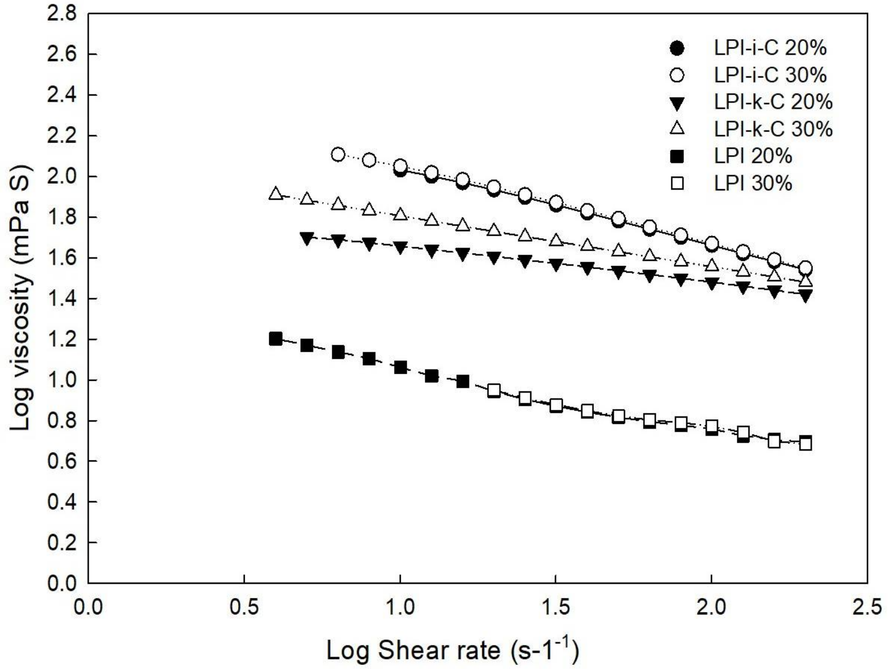

Figure S1. Flow diagram as fitted using the Power-law model parameters for emulsions stabilized by homogeneous lentil protein isolate (LPI) solutions and mixtures of LPI with κ-, λ-carrageenan at pH 6 to deliver 20% and 30% oil. Data represents the mean ± one standard deviation (n = 3). The percentage on the name of the samples represents the final oil content in dried powders.

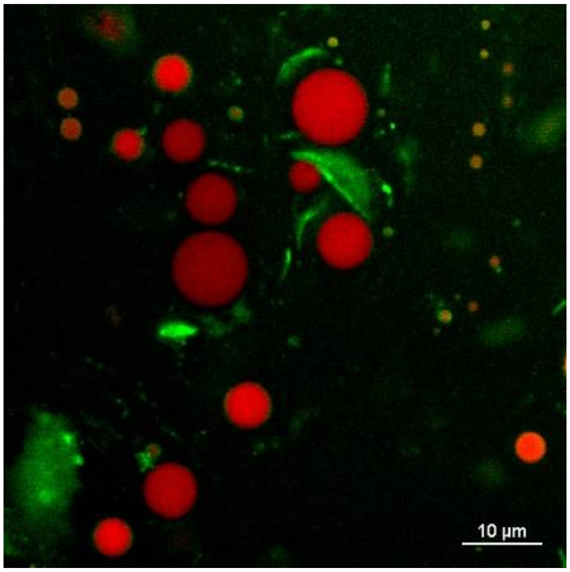

LPI- ι-C 20%

Figure S2. Confocal laser scanning microscopy (CLSM) images of a representative image for system of LPI-ι-carrageenan 20% to show the protein (green) surrounding the oil droplets (red). The scale bar represents 10 µ. The percentage on the name of the sample represents the final oil content in dried powder.

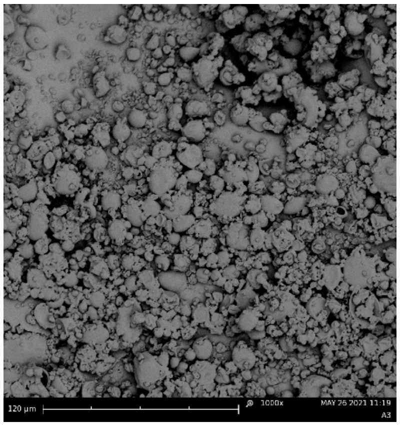

A) Spray-dried LPI- κ-C 20%

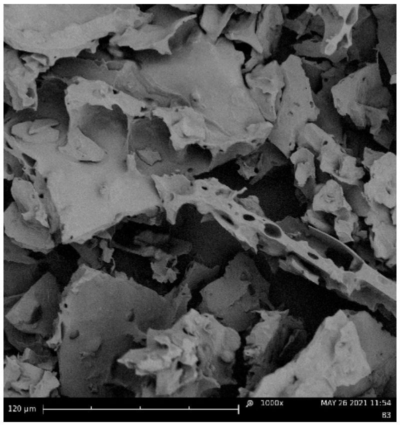

B) Freeze-dried LPI- κ-C 20%

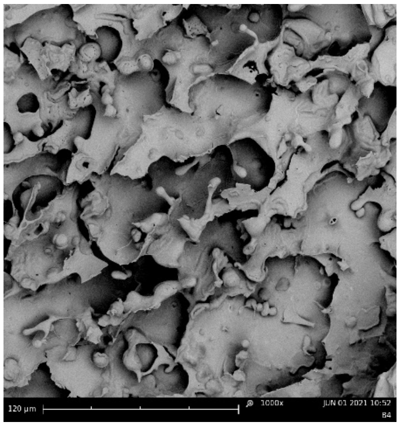

C) Freeze-dried LPI- κ-C 30%

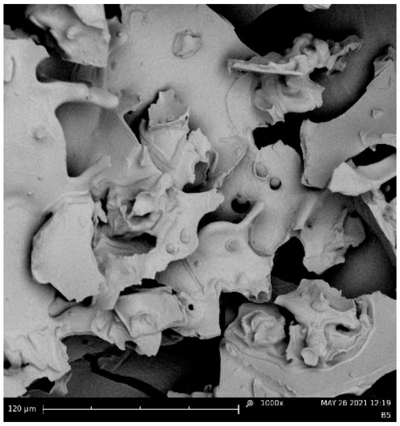

D) Freeze-dried LPI 20%

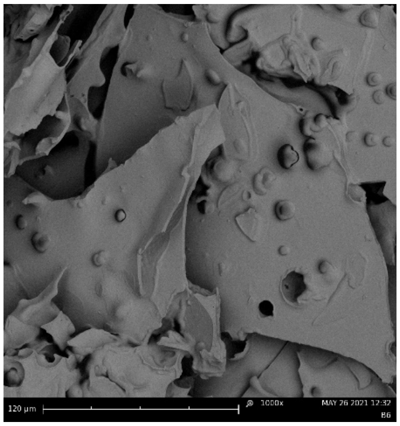

E) Freeze-dried LPI 30%

Figure S3. Scanning electron microscopy (SEM) images of the spray-dried LPI-κ-carrageenan (LPI-κ-C) 20% powder (A), freeze-dried LPI-κ-C 20% and 30% powders (B and C, respectively), freeze-dried LPI 20% and 30% powders (D and E, respectively) at 1000X magnification. All the scale bars represent 120 μ.
